# Amyand's Hernia in an Incarcerated Right Inguinoscrotal Hernia: Surgical Management of a Rare Presentation

**DOI:** 10.7759/cureus.96950

**Published:** 2025-11-16

**Authors:** Imran M Adam, Ibrahim Adhil, Abdulla Ubaid, Aishath Azna Ali, Midhuhath Rasheed

**Affiliations:** 1 General Surgery, Indira Gandhi Memorial Hospital, Malé, MDV

**Keywords:** amyand’s hernia, appendectomy, bassini repair, inguinoscrotal hernia, surgical case reports

## Abstract

Amyand’s hernia is an uncommon form of inguinal hernia in which the appendix is found within the hernia sac. Inflammation of the appendix in this setting is exceptionally rare, and the diagnosis is most often confirmed during surgery rather than before. We report a case of Amyand’s hernia presenting as an incarcerated right inguinoscrotal hernia in a 56-year-old male. Emergency surgical exploration revealed an inflamed but non-perforated appendix within the hernia sac, confirming the diagnosis. Appendectomy and hernia repair were performed using the Bassini technique, with mesh avoided due to the suspicion of contamination. The case highlights the diagnostic challenges, intraoperative findings, and surgical management considerations of this rare clinical entity. Amyand’s hernia is a rare and often unexpected intraoperative finding. Early recognition and appropriate surgical management, tailored to the condition of the appendix, are crucial to optimize outcomes.

## Introduction

Amyand's hernia is a rare type of inguinal hernia in which the vermiform appendix lies within the hernia sac. It is described after Claudius Amyand, who, in 1735, conducted the first reported successful appendectomy in an 11-year-old boy at St. George's Hospital in London [1]. The condition is uncommon, accounting for approximately 0.1% to 1% of all inguinal hernias [2,3]. Amyand’s hernia can occur at any age but is more frequently observed in male children and elderly adults [4]. It may present as either a reducible or incarcerated hernia, and the appendix itself may be normal, inflamed, or perforated. Symptoms are typically non-specific and often resemble those of a standard inguinal hernia such as groin swelling or tenderness [5].

Due to the lack of distinctive symptoms, preoperative diagnosis is rare and usually made incidentally during surgery [6]. However, imaging such as ultrasound or CT scan may occasionally identify the appendix within the hernia sac, especially in complicated cases [7,8]. Management depends on the condition of the appendix. If the appendix is inflamed or perforated, appendectomy is indicated, and mesh repair is avoided due to the risk of infection [4,9]. In cases where the appendix appears normal, some surgeons advocate for its preservation and the use of mesh repair, although this approach remains a topic of debate [10]. Amyand’s hernia underscores the importance of careful intraoperative assessment during inguinal hernia surgery. Early recognition and tailored management are key to minimizing complications [11].

## Case presentation

**Figure 1:**
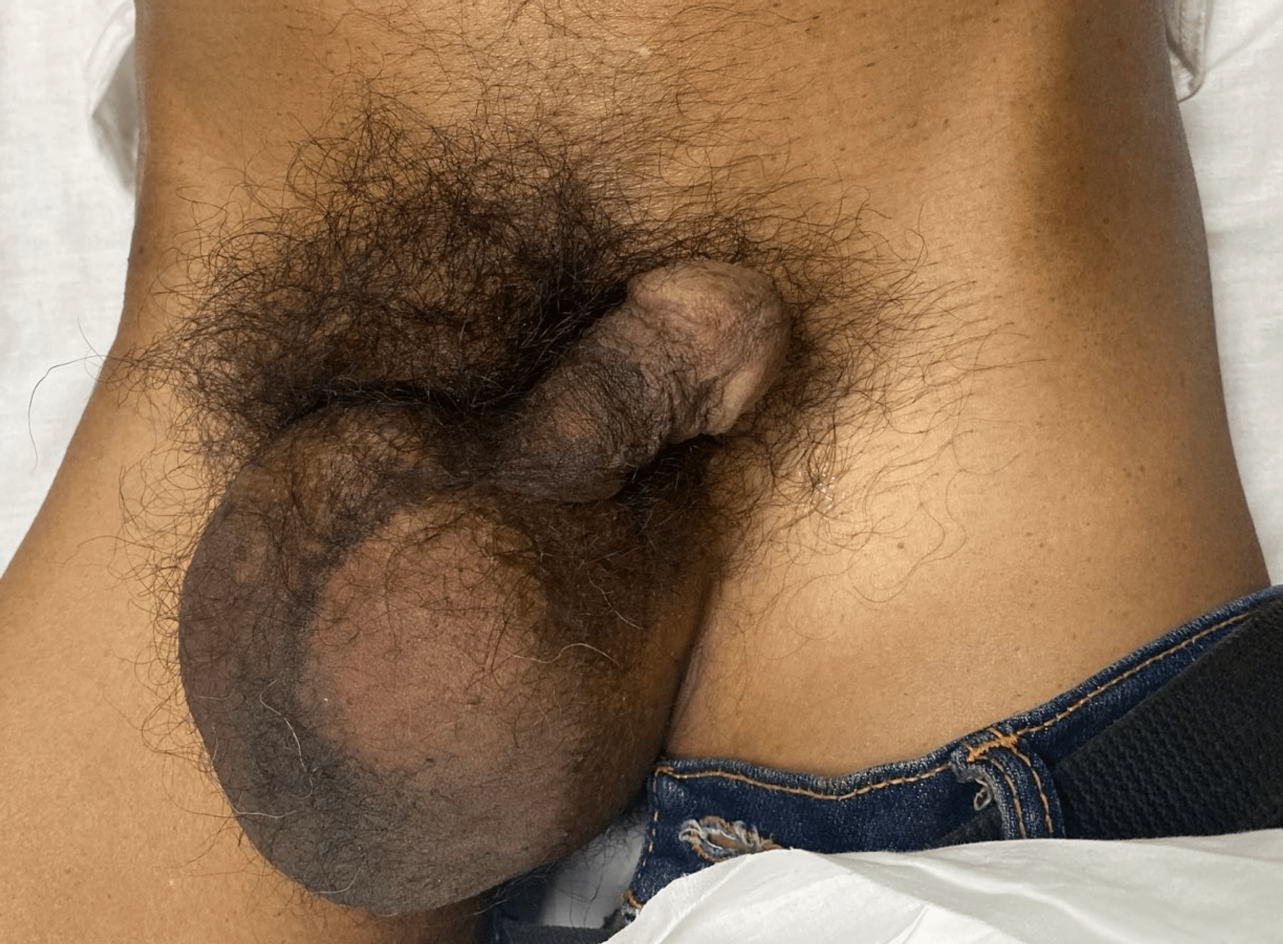
Right inguinoscrotal swelling on physical examination

A 56-year-old Maldivian male, occupied as a carpenter, with normal BMI, visited the emergency department of Indira Gandhi Memorial Hospital, Maldives, with a seven-hour duration of painful swelling in the right inguinal area (Figure 1). He had nausea but no vomiting and no acute bowel movement abnormalities. He was diagnosed with chronic obstructive pulmonary disease (COPD) one month previously, as he is presently on an inhaler and antihistamines. No relevant surgical history or family history was documented. He is a long-term smoker with 40 pack-years of smoking exposure. On examination, the patient presented with an irreducible, painful right inguinoscrotal mass. Additionally, a tenderness in the right iliac fossa was observed.

The patient was vitally stable with laboratory findings as noted in Table 1.

**Table 1:** Laboratory values of the patient ALP: alkaline phosphatase; AST: aspartate aminotransferase; ALT: alanine transaminase; GGT: gamma-glutamyl transferase; CRP: C-reactive protein; RBS: random blood sugar

Parameter	Value	Reference range
Hemoglobin	13	11.9-15.4g/dl
Blood group	O POSITIVE	
PCV	40.2	36.2-46.3%
White blood cell count	11.1	x 10^3/uL3.91-8.77
Neutrophil	68.5	40.3-74.8%
Lymphocytes	19.9	12.2-47.1%
Monocytes	6	4.4-12.3%
Eosinophils	4.4	0.0-4.4%
Basophils	0.5	0.0-0.7%
Immature granulocyte	0.75	0.0-0.6%
Platelet count	365	x10*3/uL 151.0-304.0
ALP	112	40.0-150.0 U/L
AST	41	5.0-34.0 U/L
ALT	19	0.0-55.0 U/L
GGT	25	12.0-64.0 U/L
Na+	136	136.0-145.0 mmol/L
K+	3.7	3.5-5.1 mmol/L
CRP	< 0.10	0.0-0.5 mg/dL
RBS	97	60.0-139.0 mg/dL
Appearance	Clear	
Urinary proteins, ketone bodies, nitrates, and bilirubin	Negative	
Glucose	Negative	
pH (Urine)	9	4.5-7.5
Specific gravity (Urine)	1.02	
Nitrite (Urine)	Negative	
Bilrubin (Urine)	Negative	
Urobilinogen	Normal	
Urine leucocytes	Negative	
Urine blood	Negative	
Pus cells (UR)	0.3	0.0-5.0 / HPF
Red blood cells	0.6	0.0-3.0 / HPF
Epithelial cells (Urine)	0.1	0.0-20.0 / HPF

**Figure 2:**
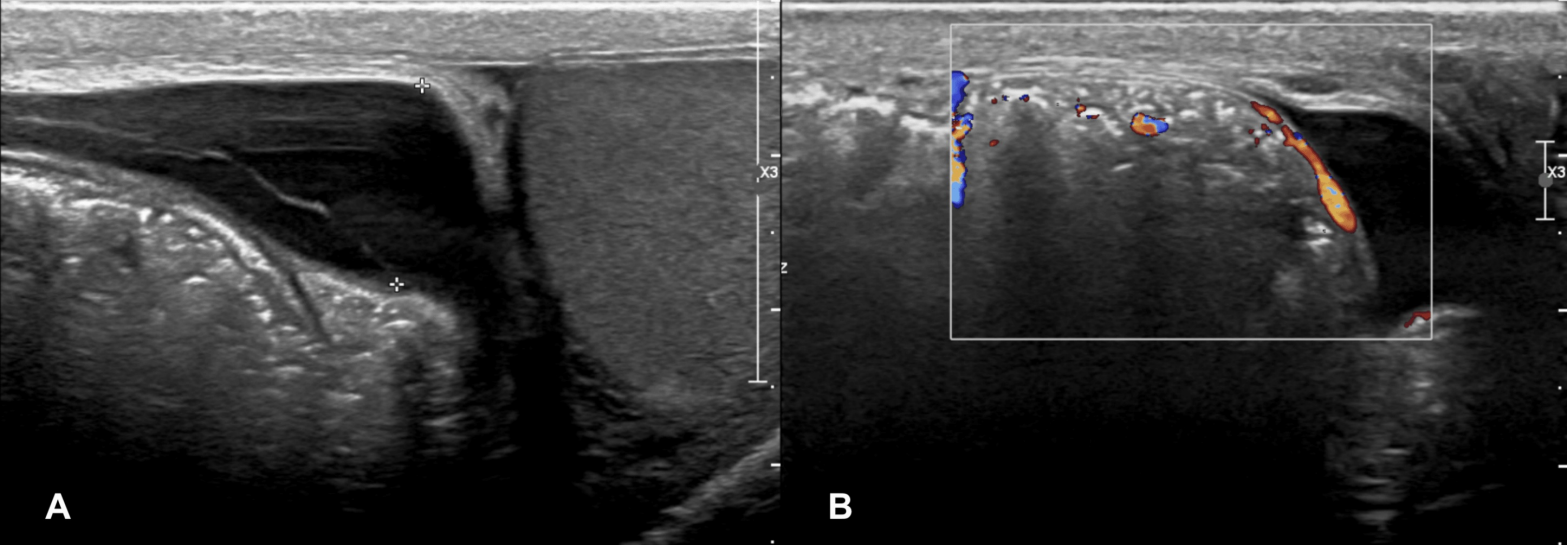
A) Ultrasound showing the right inguinoscortal hernia containing bowel loops, B) Color Doppler showing the viability of the bowel

Ultrasound revealed an incarcerated hernia with bowel loops and mesentery, but the appendix was not mentioned as a content of the hernia sac (Figure 2).

**Figure 3:**
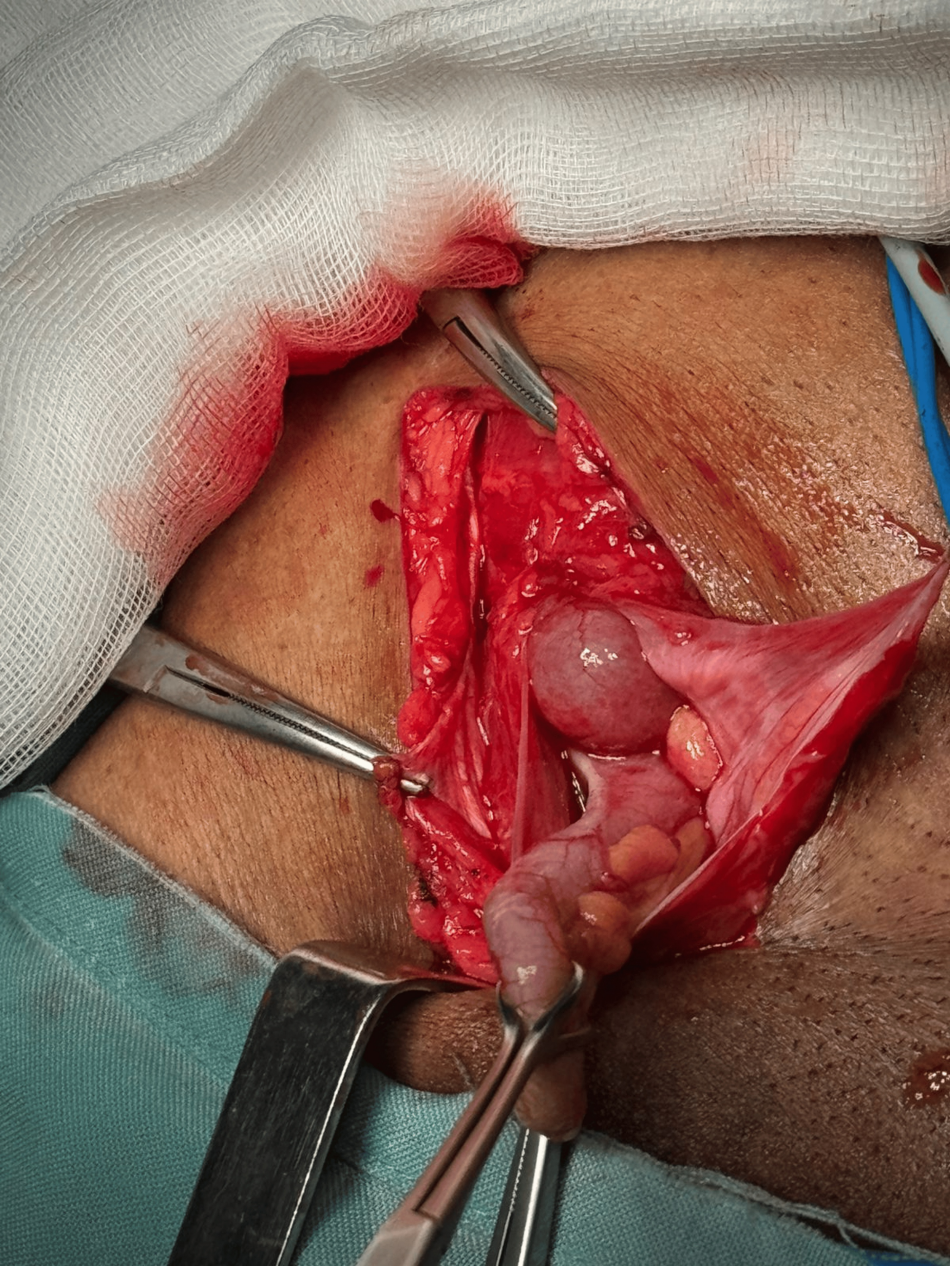
Intraoperative finding showing a non-perforated appendix within the hernia sac

**Figure 4:**
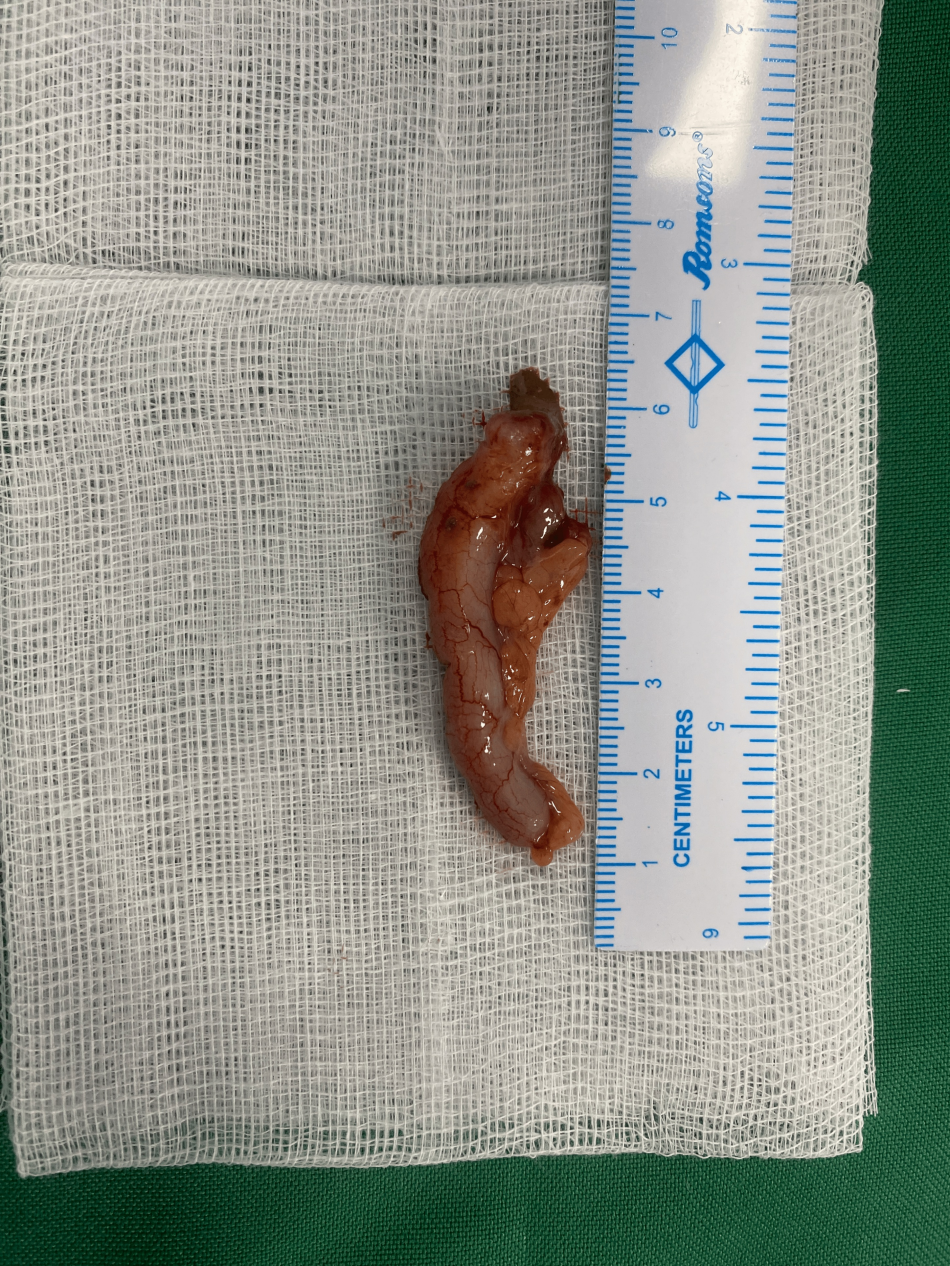
Gross specimen of the appendix following appendectomy

The patient was taken for emergency open hernia repair under general anesthesia. Intraoperatively, the hernia sac was found to contain a congested appendix, confirming a diagnosis of Amyand’s hernia (Figure 3). Appendectomy was performed, followed by the Bassini technique for the repair of the inguinal hernia (Figure 4).

The patient had an uneventful recovery and was discharged on the second postoperative day with appropriate antibiotics and analgesics.

## Discussion

Amyand’s hernia is an uncommon but clinically significant condition in which the vermiform appendix is found within an inguinal hernia sac. Although the incidence of this anomaly is reported to be around 1% of all inguinal hernias, acute inflammation of the appendix within the sac is exceedingly rare, occurring in approximately 0.1% of all cases of appendicitis [12,13]. The condition is most often encountered in males and typically affects the right side, reflecting the normal anatomical position of the appendix. Left-sided Amyand’s hernia has been described only in rare instances, usually associated with situs inversus, intestinal malrotation, or a mobile cecum.

Clinically, Amyand’s hernia often presents as a standard inguinal hernia, and the presence of the appendix within the sac may remain asymptomatic for years. Symptoms usually manifest only when appendiceal inflammation develops, leading to acute appendicitis. When this occurs within a confined hernia sac, inflammation and edema can rapidly progress to incarceration, strangulation, or even perforation [14]. These complications can result in peritonitis or scrotal abscess formation if the infection extends beyond the hernia sac. Because of this nonspecific presentation, Amyand’s hernia can easily be misdiagnosed as an incarcerated or strangulated inguinal hernia, especially when systemic symptoms are mild or absent.

Preoperative diagnosis remains a major challenge due to its rarity and the absence of distinctive clinical features. Physical examination findings are often indistinguishable from those of other incarcerated hernias. Although imaging modalities, such as ultrasound and computed tomography (CT), can theoretically identify the appendix within the hernia sac, these findings are seldom observed in practice [8,14]. Ultrasound may demonstrate a blind-ending tubular structure within the sac, while CT imaging, if performed, can show a tubular structure with wall thickening and periappendiceal fat stranding extending into the inguinal canal. However, such investigations are not routinely ordered in straightforward cases of inguinal hernia, and hence, most diagnoses are made incidentally during surgery, as occurred in our patient [15].

Losanoff and Basson proposed a practical classification system to guide the surgical management of Amyand’s hernia [9]. Type 1 is where a normal appendix is within an inguinal hernia sac, managed by hernia reduction and mesh repair without appendectomy. Type 2 involves an inflamed, non-perforated appendix within the hernia sac, which is managed through appendectomy and primary tissue repair while avoiding mesh due to the risk of contamination. Type 3 is defined as a perforated appendix with localized or generalized peritonitis requiring laparotomy, appendectomy, and hernia repair without mesh. Type 4 is an Amyand’s hernia associated with other intra-abdominal pathology; treatment is tailored to the associated disease process.

Our patient was classified as having Type 2 Amyand’s hernia, featuring an inflamed but non-perforated appendix without signs of sepsis. In such cases, appendectomy is considered the standard of care, followed by a tension-free, tissue-based hernia repair. The decision to avoid mesh is based on the well-established principle that prosthetic material should not be used in potentially contaminated surgical fields due to the high risk of infection.

We performed a Bassini repair after appendectomy, which remains one of the most reliable and reproducible tissue repair techniques in settings where mesh use is contraindicated [3]. Although prosthetic mesh repair has demonstrated lower recurrence rates in clean cases, several studies caution against its use when there is any suspicion of infection or contamination. Some surgeons advocate for a delayed or staged mesh repair once the infection has resolved [11], while others suggest biologic mesh as a potential alternative, as it carries a lower risk of infection and integrates with host tissue. However, the availability and cost of biologic materials often limit their use, particularly in resource-constrained environments.

The ongoing debate regarding mesh use in Amyand’s hernia underscores the need for individualized surgical decision-making. Factors such as the patient’s general condition, degree of inflammation, intraoperative contamination, and availability of materials all play crucial roles in determining the optimal approach. Moreover, laparoscopic approaches have been described in select cases, especially when the diagnosis is made preoperatively or in recurrent hernias. Laparoscopy allows both diagnostic confirmation and treatment, including appendectomy and hernia repair through minimally invasive means, although its role remains limited to experienced centers.

Postoperative outcomes in Amyand’s hernia are generally favorable when timely diagnosis and appropriate management are achieved. Delayed intervention, on the other hand, can lead to complications such as wound infection, abscess formation, or hernia recurrence. In our case, the patient had an uneventful recovery following appendectomy and Bassini repair, reflecting the importance of early recognition and adherence to established surgical principles.

In summary, Amyand’s hernia represents a rare but important diagnostic and surgical entity. Surgeons should maintain a high index of suspicion when encountering atypical or irreducible inguinal hernias, particularly those with signs of inflammation. Although preoperative diagnosis remains difficult, intraoperative vigilance and tailored management according to the Losanoff and Basson classification can significantly improve patient outcomes. Tissue-based repair remains the preferred technique in contaminated fields, while mesh repair can be safely reserved for clean cases or delayed until after infection has resolved.

## Conclusions

Amyand’s hernia remains a rare but important consideration in cases of incarcerated inguinoscrotal hernias. Because preoperative diagnosis is difficult, high suspicion and intraoperative adaptability are important for proper treatment. Surgical planning must be adapted to intraoperative results. Awareness and prompt intervention are key to achieving favorable outcomes in such rare presentations.
